# Multi-Agent System Based Cooperative Control for Speed Convergence of Virtually Coupled Train Formation

**DOI:** 10.3390/s24134231

**Published:** 2024-06-29

**Authors:** Chuanzhen Liu, Zhongwei Xu

**Affiliations:** 1 School of Electronic and Information Engineering, Tongji University, Shanghai 201804, China; liuchuanzhen88@163.com; 2 Shanghai Hengjun Technology Co., Ltd., Shanghai 200949, China

**Keywords:** train-formation cooperative control, multi-agent systems, distributed observer, barrier Lyapunov function, speed convergence

## Abstract

This paper investigates the problem of spacing control between adjacent trains in train formation and proposes a distributed train-formation speed-convergence cooperative-control algorithm based on barrier Lyapunov function. Considering practical limitations such as communication distance and bandwidth constraints during operation, not all trains can directly communicate with the leader and obtain the expected trajectory it sends, making it difficult to maintain formation consistency as per the predetermined ideal state. Furthermore, to address the challenge of unknown external disturbances encountered by trains during operation, this paper designs a distributed observer deployed on each train in the formation. This observer can estimate and dynamically compensate for unknown reference trajectories and disturbances solely based on the states of adjacent trains. Additionally, to ensure that the spacing between adjacent trains remains within a predefined range, a safety hard constraint, this paper encodes the spacing hard constraint using barrier Lyapunov function. By integrating nonlinear adaptive control theory to handle model parameter uncertainties, a barrier Lyapunov function-based adaptive control method is proposed, which enables all trains to track the reference trajectory while ensuring that the spacing between them remains within the preset interval, therefore guaranteeing the asymptotic stability of the closed-loop system. Finally, a practical example using data from the Guangzhou Metro Line 22, specifically the route from Shiguang Road Station to Chentougang Station over three stations and two sections, is utilized to validate the effectiveness and robustness of the proposed algorithm.

## 1. Introduction

Urban rail transit undertakes the task of transporting millions, or even tens of millions, of passengers daily, serving as the “arteries” of urban public transportation and playing a crucial role in alleviating urban traffic congestion [1,2,3,4,5,6,7,8,9]. However, with the continuous expansion of urban areas, phenomena such as peak-hour passenger flows caused by the separation of work and residence and sudden surges in passenger flows due to large-scale events often lead to passenger congestion in local areas and station crowding, resulting in a decrease in the transportation capacity of the rail network or even paralysis, severely affecting the operational quality and passenger experience of urban rail transit. Faced with the unbalanced demand for capacity caused by peak-hour and sudden passenger flows, the current widely adopted moving-block train operation control systems for urban rail transit have reached the limits of their designed operational capacity [10]. The capacity of existing lines has reached saturation, and it is not feasible to achieve rapid transport of large-scale passenger flows by increasing the density of trains in local areas [11]. Moreover, factors such as the high cost of building new lines and constraints imposed by limited spatial resources make it difficult to meet diverse travel demands. Therefore, improving transportation capacity, achieving flexible matching of capacity and passenger flows, and meeting actual passenger transportation needs are the main research areas in the field of urban rail transit at present.

With the continuous advancement of rail transit technologies such as train-to-train communication and Automatic Train Operation (ATO), considerable attention has been directed towards Train Formation (also known as virtual coupling) technology [4,12,13]. In existing schemes, physical couplings between trains are no longer employed; instead, formations are established through inter-train communication, wherein trains exchange information to operate collectively in a coordinated manner. Based on information received from other trains within the formation, onboard systems are tasked with tracking target velocities while maintaining inter-train distances, enabling trains to follow preceding ones at safe intervals. This approach significantly reduces inter-train gaps, therefore notably enhancing line throughput capacity. Konig et al. elucidated the fundamental concepts of train formation, proposed innovative operational principles and key technological requirements pertaining to communication, positioning, and control within train formations, devised a system simulation model, and validated the effectiveness of the aforementioned functionalities [14]. Subsequently, with the introduction of the European Union’s “Shift2Rail” [15] and “Horizon 2020” [16] research initiatives, train-formation technology has once again become a fresh focal point within the industry. Flammini et al. contextualized their research against the backdrop of ERTMS/ETCS, focusing on the principal advantages, current obstacles, and future contexts of train-formation technology. They proposed the notion of integrating train-formation technology to extend the current ERTMS-3 standard [17], utilizing existing infrastructure and onboard equipment. Furthermore, they analyzed the safety and reliability of this approach and provided recommendations for maintaining backward compatibility of equipment [18]. Subsequently, Aoun et al. delved into the specific requirements of railway signaling technology for train formation from safety, technical, and operational perspectives. They defined a set of new operational scenarios based on train formation to assess its advantages in enhancing capacity. Muniandi et al. conducted extensive work on train-formation strategies, employing simulation and analysis with examples to validate the efficacy of their proposed methods [19,20,21,22,23,24]. However, despite the various attempts from different angles to address the collaborative control of train formations, the coordination of train formations during speed-convergence stages remains relatively intricate, necessitating a thorough elucidation of the operational processes of train formations, an aspect yet to be investigated.

Formation control has always been a focal research issue in the field of multi-agent coordination. It refers to the control problem where a system composed of multiple agents maintains a predetermined geometric formation (i.e., formation) while moving towards specific targets or directions and adapts to environmental constraints (such as avoiding obstacles) [25,26]. Researchers such as K. categorized the results of formation control studies into three types based on different perceptual capabilities and interaction topologies of agents: position-based, displacement-based, and distance-based control. They detailed the consensus protocol frameworks under these three control modes, providing a clear overview of multi-agent formation control [27]. Chen et al. mainly focused on achievements in the fields of unmanned vehicle formation and robot formation, elucidating stability and controllability analysis methods for formation control and introducing formation control strategies based on behavior and potential functions, leader-follower methods, generalized coordinates, and virtual structure methods [28]. Liu et al. proposed a distributed nonlinear control method for a multi-agent system with variable directed topologies and speed constraints, where only a non-stringent connectivity condition needs to be satisfied to ensure the gradual convergence of controlled agent outputs [29]. Subsequently, fruitful research results have been achieved in the field of multi-agent formation control for near-Earth satellite formation flight, robot formation, and unmanned aerial vehicle formation. In recent years, some scholars have introduced the theory of multi-agent formation control into the field of train control by analogy with vehicle formation. Scholars such as Song modeled the problem of dynamic adjustment of train headway as a multi-agent formation control problem, introduced artificial potential functions, and proposed a real-time dynamic headway adjustment algorithm. Simulation results demonstrated the robustness of this method against disturbances [30]. Zhao et al. studied the cruise control problem for single high-speed trains with multiple carriages and multiple high-speed train columns. They designed a universally applicable formation train control consensus protocol by selecting appropriate traction nodes and demonstrated the effectiveness of the algorithm [31]. Although there are few reports on train-formation control problems based on multi-agent systems, they are still in the preliminary theoretical exploration stage and have not addressed specific application scenarios of train-formation. Researching how to effectively solve train-formation control problems using methods based on multi-agent system formation control is the primary focus of this paper.

At another frontier, significant progress has been achieved in the research on train-formation control issues with the development of information technology and distributed control techniques [32,33,34,35,36]. For instance, a model predictive control (MPC) framework for train-formation control was proposed by Felez et al., wherein nominal MPC controllers were individually designed for the front train and the other trains within the formation. Simulation results demonstrate a significant reduction in headway and inter-train distances compared to the moving-block system [37]. Luo et al. devised an MPC-based train-formation control strategy by considering input constraints (velocity and acceleration constraints) and output constraints (safe headway constraints), demonstrating the asymptotic stability and string stability of the control system [38]. Additionally, Luo et al. introduced a distributed train-formation adaptive MPC control method; however, this control method did not account for unknown disturbances in the system, leading to severe oscillations in the simulation results [39]. However, the aforementioned literature fails to consider the uncertainty of train dynamic model parameters within the train formation or the uncertain disturbances present in the track. Wu et al. addressed this by proposing an MPC-based train-formation control method considering constraints such as track speed, collision avoidance, maximum traction/braking force, and train string stability, with inter-train distance error, velocity error, and passenger comfort as optimization objectives [40]. Li et al. proposed a collaborative control strategy based on a multi-agent model for the coordinated control of multiple high-speed trains [41]. Building upon this research, they proposed an adaptive collaborative control algorithm considering input saturation and parameter uncertainty [42]. It should be noted that all the aforementioned studies implicitly assume that all trains can communicate with the front train. However, due to limitations such as communication distance and bandwidth constraints, not all trains can directly communicate with the leader and receive the desired trajectory it sends. In practice, maintaining formation consistency in accordance with the predetermined ideal state is challenging. Therefore, considering the partial availability of trajectory reference information, along with the influence of disturbances and parameter uncertainties on running trains, as well as various constraints such as track speed limits, designing a distributed controller based on multi-agent system theory is of paramount importance in addressing train-formation control issues. This constitutes the second motivation of this paper.

Based on the analysis above, this paper presents a distributed observer that dynamically compensates for the system based solely on the states of adjacent trains. Additionally, to address the safety hard constraint of maintaining the spacing between adjacent trains within a predefined range and the uncertainty of train parameters, an adaptive control algorithm based on barrier Lyapunov function is designed. This algorithm ensures that the spacing between adjacent trains within the formation remains stable within the safety range. The main contributions of this paper can be summarized as follows:

- To address the issue where trains in a train formation cannot communicate with the leader train to obtain desired trajectory information due to communication distance and other limitations, this study proposes a distributed observer. Compared with existing control strategies [7,8,10,11,33,43], the proposed control approach is more effective since the distributed observer could provide a real-time dynamic compensation for the closed-loop system while only requiring the state information from adjacent trains.
- A barrier Lyapunov function-based adaptive control algorithm is proposed by transforming the requirement for maintaining the spacing between adjacent trains within a predefined safe range into a hard constraint while accounting for the parameter uncertainties introduced by train motion. By utilizing the constraint enforcement properties of the barrier Lyapunov function, the designed control algorithm theoretically can ensure that the spacing between adjacent trains in the formation remains consistently within a safe range.
- Aiming at the high safety requirements of train-formation operation, unlike the existing literature [8,11,44] which ignores the practical presence of external disturbances or system uncertainties in practice, this article models the cooperative-control problem of train formations as a leader-following aggregation problem with parameter and disturbance uncertainties, intending to maintain the spacing between adjacent trains during the speed-convergence operation phase of train formations within a safe range. Through the utilization of the nonlinear adaptive control strategy, this article effectively handles the parameter uncertainties within the train-formation model, which not only significantly reduces the stringent requirements for the accuracy of actual train-formation model parameters but also markedly enhances the robustness and adaptability of the cooperative-control algorithm.

## 2. Problem Description and Preliminaries

### 2.1. Graph Theory

A system with *n* agents is typically modeled as an *n*-order graph. Specifically, agents are represented as nodes in the graph, while the interactions between agents, arising from factors such as sensing and communication, are depicted as edges in the graph.

Let G=(V,ϵ,A) denote a directed graph of order *n*, where *V* represents the set of nodes defined as V={1,2,…,n}, and the edge set ϵ={(i,j)|i,j∈V,i≠j}⊆V×V, where an edge is described by an ordered pair of nodes (i,j). If (i,j)∈ϵ, then node *i* is considered a neighbor of node *j* if and only if there exists a path from node *i* to node *j*. The set of neighbors of the *i*-th agent in the system is denoted as $N_{i}=\left\{j|\left(j,i\right)\in \epsilon \right\}$. Let $A=\left[a_{ij}\right]\in R^{n\times n}$ denote the adjacency matrix of an *n*-order graph *G*. $a_{ij}=w$ if and only if (j,i)∈ϵ (i.e., $j\in N_{i}$), where *w* represents the weight value of the edge, otherwise, $a_{ij}=0$, i.e.,

(1) $$A={\left[a_{ij}\right]}_{n\times n},a_{ij}=\left\{\begin{array}{c} w,(j,i)\in \epsilon \\ 0,(j,i)\notin \epsilon \end{array}\right.$$

This paper assumes that the communication relationships between all nodes have the same priority, thus setting the weight value *w* to 1.

Next, the concept of a degree is introduced. For any node v∈V of directed graph *G*, the sum of the number of times *v* serves as the starting point of an edge is called the out-degree of node *v*, denoted as $d^{+}\left(v\right)$; the sum of the number of times *v* serves as the endpoint of an edge is called the in-degree of node *v*, denoted as $d^{-}\left(v\right)$; $d^{+}\left(v\right)+d^{-}\left(v\right)$ is referred to as the degree of node *v*, denoted as d(v). The degree matrix *D* stores the degrees of each node in the graph, and it is evident that the degree matrix is a diagonal matrix. The Laplacian matrix *L* of a graph is defined as the difference between the degree matrix and the adjacency matrix, i.e., L=D−A, $L=[l_{ij}]$, where $l_{ij}$ is given by $l_{ij}=\left\{\begin{array}{c} \sum_{j=1}^{n}a_{ij},\mathrm{if}\;\;i=j\\ -a_{ij},\mathrm{if}\;\;i\neq j \end{array}\right.$. The *i*-th row of *L* reflects the cumulative gain produced by the *i*-th node when interacting with all other nodes. Undirected graphs can be regarded as directed graphs with certain properties. Specifically, if a directed graph G={V,ϵ,A} simultaneously satisfies the properties (i,j)∈ϵ and (j,i)∈ϵ, then *G* can be considered to be an undirected graph.

The multi-agent system is employed to model the train formation in which each train is considered to be an agent, and the rear train of the adjacent train is able to obtain state information, such as speed, position, etc., from the front train. Therefore, one has the following assumption.

**Assumption** **1.** *The structure of a train formation can be revealed through a directed connected graph, where the vertex indicates the train and the edge represents the adjacency relationship.*

**Lemma** **1** ([45]). *All non-zero eigenvalues of the Laplacian matrix L of graph G (if they exist) have positive real parts. Furthermore, L is nonsingular if and only if the directed graph contains a directed spanning tree rooted at a non-zero node.*

### 2.2. Train Dynamics Model

**Figure 1:**
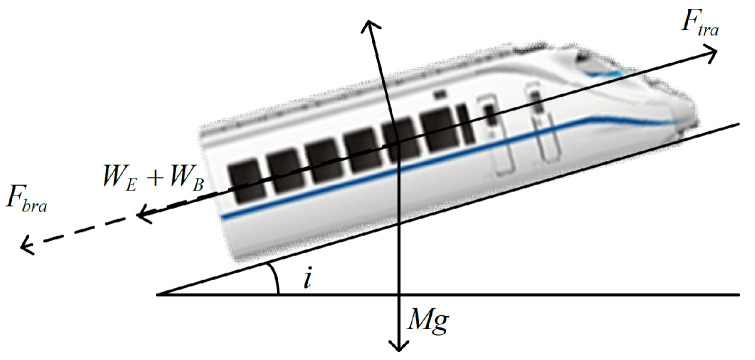
Forces analysis of a train.

Disregarding details such as the length of the train and treating it as a point mass and neglecting internal forces like inter-carriage interactions, trains typically experience traction/braking forces, rolling resistance, aerodynamic drag, as well as gradient and curve resistance. Considering the single train model depicted in Figure 1, the dynamic equation can be simplified as follows:

(2) $$\left\{\begin{array}{cc} \dot{x}\left(t\right) & =v(t)\\ M\dot{v}\left(t\right) & =F\left(t\right)-M\left(c_{0}+c_{1}v\left(t\right)+c_{2}v^{2}\left(t\right)\right)-W_{E}+D\left(w\right) \end{array}\right.$$

where *x* represents the train’s position (m); *v* represents the train’s velocity (m/s); *F* represents the traction/braking force (N) applied to the train, where $F_{tra}$ denotes traction force and $F_{bra}$ denotes braking force; *M* represents the mass of the train (kg); $c_{0},c_{1}$ and $c_{2}$ are coefficients used to calculate the Davis equation coefficients of basic resistance $W_{B}$, typically derived from empirical statistics, where $c_{0}\left(N/\mathrm{kg}\right)$ comprises rolling friction and bearing friction, $c_{1}\left(N/m/\mathrm{kg}\right)$ is the resistance coefficient, usually a positive value, and $c_{2}\left(N^{2}/m^{2}/\mathrm{kg}\right)$ is the aerodynamic resistance coefficient; D(w) represents unknown disturbances during train operation; $W_{E}$ represents additional resistance experienced by the train, composed of the incline additional resistance $W_{i}$ and the curve additional resistance $W_{r}$, where the unit incline additional resistance is denoted as $w_{i}=i$ and i(%) is determined by the slope gradient, positive for uphill and negative for downhill; the unit curve additional resistance $w_{r}=600/R$ is determined by the curve radius R(m).

Since the track slope *i* and the curve radius *R* only depend on the location of the train on the track, they are known at any given time during the train operation. Therefore, (2) can be rewritten as:

(3) $$\left\{\begin{array}{cc} \dot{x}\left(t\right) & =v(t)\\ \dot{v}\left(t\right) & =\frac{u(t)}{M}-\left(c_{0}+c_{1}v\left(t\right)+c_{2}v^{2}\left(t\right)\right)+d\left(w\right) \end{array}\right.$$

where d(w) the unit unknown disturbance during train operation; u(t) represents the train control input.

### 2.3. Dynamic Model of Train Formation

**Figure 2:**
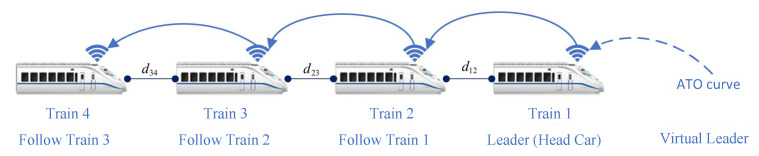
Operation model of train formation.

As shown in Figure 2, for train formation, the dynamics equation is formulated as follows:

(4) $$\left\{\begin{array}{cc} {\dot{x}}_{i}\left(t\right) & =v_{i}\left(t\right)\\ {\dot{v}}_{i}\left(t\right) & =\frac{u_{i}\left(t\right)}{M_{i}}-\left(c_{i0}+c_{i1}v_{i}\left(t\right)+c_{i2}v_{i}^{2}\left(t\right)\right)+d_{i}\left(w\right) \end{array}\right.$$

where $x_{i}\left(t\right)$ is the current position of the *i*-th train within the train formation (m); $v_{i}\left(t\right)$ represents the current velocity of the *i*-th train within the train formation (m/s); $M_{i}$ stands for the mass of the *i*-th train within the train formation (kg); $c_{i0},c_{i1},c_{i2}$ are the basic resistance coefficients of the *i*-th train within the train formation; $d_{i}\left(w\right)$ is the unit disturbance experienced by the *i*-th train within the train formation during operation.

**Remark** **1.** *The basic resistance coefficients $c_{i0}$, $c_{i1}$, and $c_{i2}$ in the Davis equation are typically derived from empirical formulas. Variations in train parameters due to factors such as aging can lead to significant uncertainties in the actual basic resistance coefficients of each train within the train formation compared to their nominal values. Additionally, various noise interferences during train operation cannot be directly measured. These factors lead to model mismatch, which can result in poor control performance and even amplify deviations from the equilibrium point along the train formation.*

### 2.4. Analysis of Train-Formation Operation Process

The operation of train formation involves three main processes: dynamic formation establishment, speed convergence, and dynamic dissolution. The minimum safe distance between trains is defined as $R_{2}$, and the maximum communication range is $R_{1}$, with a constant γ belonging to the interval $\left(0,R_{1}\right)$.

**Figure 3:**
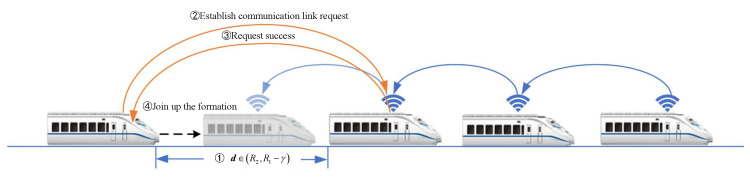
Diagram of coupling process for virtually coupled train formation.

**Figure 4:**
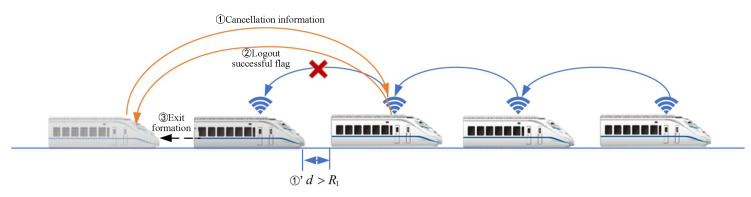
Diagram of decoupling process for virtually coupled train formation.

**Figure 5:**
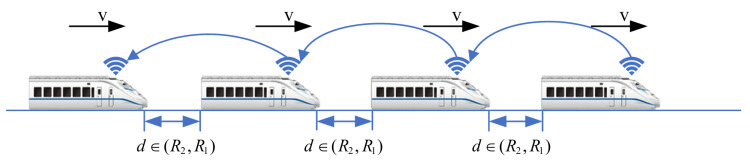
Diagram of speed convergence of virtually coupled train formation.

1. Dynamic Formation Establishment Process: Assuming a train *i* not belonging to the formation is within the range of $\left(R_{2},R_{1}-\gamma \right)$ with respect to the tail train of the formation in Figure 3, train *i* requests to establish a communication link with the tail train. Upon successful establishment, train *i* joins the formation, and this process repeats until all trains within the scheduled route have joined the formation. Upon completion of the dynamic formation establishment process, the train formation enters the speed-convergence process.
2. Train-Formation Disaggregation Process: If the distance between train *i* and the preceding train exceeds the maximum communication distance $R_{1}$, or if train *i* loses communication directly with the preceding train, and if the communication link is not reestablished within the specified timeframe, train *i* and its subsequent trains will withdraw from the formation in Figure 4.
3. Train-Formation Speed-Convergence Operation Process: As shown in in Figure 5, the process of speed convergence within train formations is the primary focus of this article. Through inter-train communication, trains within the formation acquire status information such as speed and position from the front train. This information is then compared with the corresponding status of the local train. Utilizing a cooperative-control algorithm, the speed of adjacent trains is synchronized while maintaining a prescribed distance between them. In other words, trains within the formation achieve synchronized travel at identical speeds through cooperative control. Meanwhile, the spacing between adjacent trains remains stable within the range of $\left(R_{2},R_{1}\right)$, ensuring collision-free operation throughout the journey.

### 2.5. Collaborative Control Objectives for Train Formation

In a train formation, the leading train (Train 1) tracks the ATO curve for operation. Meanwhile, other trains within the formation (Train 2, 3, 4, etc.), also known as follower trains, establish communication links with adjacent trains through the inter-train communication system. They continuously receive real-time status information, including speed and position, from the front train. Each follower train regards the front train as the leader and utilizes the ATO curve tracked by the front train as the reference trajectory. By utilizing the route information stored in the database and the local status information, each follower train calculates and controls itself to track the reference trajectory, as well as regulates the tracking distance between adjacent trains to achieve the overall control objective of the formation, i.e., ensure that collision-free operation for all trains within the formation and ultimately achieve synchronized travel at the same speed for the entire formation.

Due to the complexity of the operating environment of urban rail transit train formation, the entire working process of train formation is subject to various sources of noise and disturbances. Moreover, uncertainties arise in the train formation’s dynamic model due to factors such as the train’s aging, which leads to parameter deviations. Additionally, the trains in the formation are subject to various physical constraints during operation while needing to ensure their operational safety and performance. This requires some constraints to be considered in the design of the cooperative-control algorithm, such as the range of traction/braking forces. Given the above discussion, the research objectives of this chapter regarding the cooperative-control algorithm for train formation are outlined as follows: (1) Considering the safety and performance requirements during the operation, it is imperative to ensure that the spacing between adjacent trains within the train formation consistently exceeds the minimum safety distance during the speed-convergence phase. Moreover, efforts should be made to maintain this spacing as close as possible to the ideal distance; (2) By designing a distributed cooperative-control algorithm for train formation, the objective is to achieve consistency in speed tracking during the operation of the train formation; (3) The designed distributed cooperative-control algorithm for train formation should exhibit robustness and stability, enabling the train formation to overcome the uncertainties arising from model inaccuracies and disturbances during the speed-convergence phase of the cooperative-control process.

The control objective of train formation indicates that the cooperative-control problem of speed convergence essentially constitutes a regulation (stabilization) problem. Each train (i=2,…,n) within the formation regards the front train (i=2) as the leader. The ATO curve tracked by the front train serves as the reference trajectory for the entire formation, ensuring that the entire train formation eventually converges to the same operating speed while maintaining stable spacing between adjacent trains within a preset interval. By analyzing the above control objectives through the lens of multi-agent theory, the problem of cooperative control for speed convergence in train formations can be modeled as a leader-following aggregation problem. Essentially, it represents a regulation problem for a multi-agent system with reference signals and disturbances, where the states (or partial states) of all agents track the desired trajectory generated by a leader system. The reference trajectory can be considered to be the virtual leader of the system.

**Remark** **2.** *Please note that in practical operation, various factors, such as limited communication distance and resources, restrict the condition where all trains within the train formation can directly communicate with the leader, which tracks the ATO curve, serving as the reference trajectory for the entire formation. Only the front train within the formation can access this ATO curve. Other trains within the formation can only communicate with adjacent trains, relying on real-time status information from neighboring trains and their own status to control their behavior in real-time.*

**Remark** **3.** *As stated in Remark 1, there is significant uncertainty in the basic drag coefficients of trains within the train-formation model compared to their nominal values in practical operation. Moreover, the external disturbances experienced during the operation of the train formation are typically unknown and difficult to measure directly. To achieve effective control performance, it is essential to consider the influence of these uncertainties in the design of the control algorithm for train formations.*

Consider a formation consisting of *n* trains, where the kinematic equation for any arbitrary train *i* within the formation is represented as (4). Let ${\bar{u}}_{i}\left(t\right)=\frac{u_{i}\left(t\right)}{M}$ be the unit control input to be designed for train *i*, and $\theta_{i}={\left[\begin{array}{ccc} c_{i0} & c_{i1} & c_{i2} \end{array}\right]}^{T}$ be a vector of unknown constant parameters. Thus, Equation (4) can be rewritten as:

(5) $$\left\{\begin{array}{cc} {\dot{x}}_{i}\left(t\right)= & v_{i}\left(t\right)\\ {\dot{v}}_{i}\left(t\right)= & {\bar{u}}_{i}\left(t\right)-\left[\begin{array}{ccc} 1 & v_{i}\left(t\right) & v_{i}^{2}\left(t\right) \end{array}\right]\theta_{i}+d_{i}\left(w\right) \end{array}\right.$$

where *w* represents a disturbance factor, which is an uncertain parameter. Let $x_{r}\left(t\right)$ and $v_{r}\left(t\right)$ denote the expected position and expected velocity in the reference trajectory of the train formation, respectively. For the class of multi-agent system regulation problems, such as the cooperative-control problem for train-formation speed convergence involving reference signals and disturbances, the disturbances and reference trajectory are commonly combined and referred to as external signals, assumed to be generated by an external system. Let us denote the external signals of the train-formation system as:

(6) $$\begin{array}{c} o=\left[\begin{array}{ccc} x_{r}\left(t\right) & v_{r}\left(t\right) & w \end{array}\right] \end{array}$$

The external system is represented by

(7) $$\begin{array}{c} \dot{o}=Co \end{array}$$

Next, for the sake of convenience in derivation, we introduce the following reasonable assumptions:

**Assumption** **2.** *The function distribution $d_{i}\left(•\right)$ to which the unknown disturbance $d_{i}\left(w\right)$ belongs is a function of some $C^{1}$ class, which is continuously differentiable over its definition domain while having continuous first-order partial derivatives.*

**Assumption** **3.** *The external system (7) is neutrally stable.*

**Assumption** **4.** *For the train-formation system represented by Equation (4), when t=0, the communication topology G of the system is connected, and the initial states of each train do not violate any constraints.*

**Remark** **4.** *Actual train formation is inevitably subject to disturbances in the external environment during operation. For a certain type of persistent external disturbance, such as mechanical wear and tear leading to actuator efficiency degradation or wind resistance variation due to different weather, although the disturbance itself is unknown, one can judge that it can be quantitatively described as a continuously varying functional form according to the common sense of physics and the partial derivatives concerning its independent variables exist as well as being continuous. Thus, Assumption 2 is reasonable and easily satisfied.*

The primary objective of this article is to design an adaptive distributed controller for the train formation (4) and the external system (7). Given any specified maximum communication range between adjacent trains $R_{1}>0$ and the minimum safety distance between trains $R_{2}\in \left[0,R_{1}\right)$, the aim is to ensure, under the Assumptions 2–4, that the closed-loop system, with given initial states and parameters, satisfies:

(1) The spacing between any adjacent trains within the train formation remains stable within the interval $\left(R_{2},R_{1}\right)$ throughout the entire operation, ensuring that the communication topology of the train formation remains connected and preventing collisions between adjacent trains.
(2) All trains within the formation can track the reference velocity $v_{r}$, therefore fulfilling the following consistency protocol:
  (8) $$\begin{array}{c} \lim_{t\to \infty }\left(v_{i}-v_{r}\right)=0 \end{array}$$

### 2.6. Observer Design

To compensate for the external system, inspired by the dynamic compensator for addressing the collaborative output regulation problem in linear multi-agent systems [46], we design a distributed observer as follows:

(9) $$\begin{array}{c} {\dot{\hat{o}}}_{i}=C{\hat{o}}_{i}+\mu \sum_{j=0}^{n}a_{ij}\left({\hat{o}}_{j}-{\hat{o}}_{i}\right),i=1,\cdots ,n \end{array}$$

where μ is a positive constant to be designed; ${\hat{o}}_{i}$ is the estimate of the vector $o_{i}$, where $o_{i}=\left[\begin{array}{ccc} x_{t} & v_{r} & w \end{array}\right]$; *C* is the observation matrix.

From (9), it is evident that each train within the formation can dynamically compensate for the state of the virtual leader (i.e., the reference signal) and the disturbance experienced by this train solely based on the state information of neighboring trains.

### 2.7. Controller Design

For the convenience of subsequent analysis, we define the error between the position of any train *i* within the formation and the reference position as ${\hat{x}}_{i}\left(t\right)=x_{i}\left(t\right)-x_{r}\left(t\right)$, and the velocity error as ${\hat{v}}_{i}\left(t\right)=v_{i}\left(t\right)-v_{r}\left(t\right)$. In the following discussions, the notions $x_{i}$ and $v_{i}$ are used to denoted $x_{i}\left(t\right)$ and $v_{i}\left(t\right)$ for simplicity.

To handle the spacing constraints between adjacent trains in the train-formation system, we first construct a barrier Lyapunov function, which is selected as:

(10) $$\begin{array}{c} V\left(d_{ij}\right)=\frac{1}{{\left(R_{1}^{2}-d_{ij}^{2}\right)}^{2}}+\frac{1}{{\left(d_{ij}^{2}-R_{2}^{2}\right)}^{2}} \end{array}$$

where $R_{1}$ and $R_{2}$ represent the maximum communication distance and the minimum safety distance between adjacent trains within the formation.

**Remark** **5.** *It should be noted that as the distance between trains i and j gradually increases and approaches $R_{1}$, indicating that trains i and j are on the verge of losing communication, the value of the barrier Lyapunov function $V(d_{ij})$ tends to infinity. Conversely, if the distance between trains i and j decreases gradually until it approaches $R_{2}$, implying an imminent collision between the trains, the value of the barrier Lyapunov function $V(d_{ij})$ also tends to infinity.*

**Figure 6:**
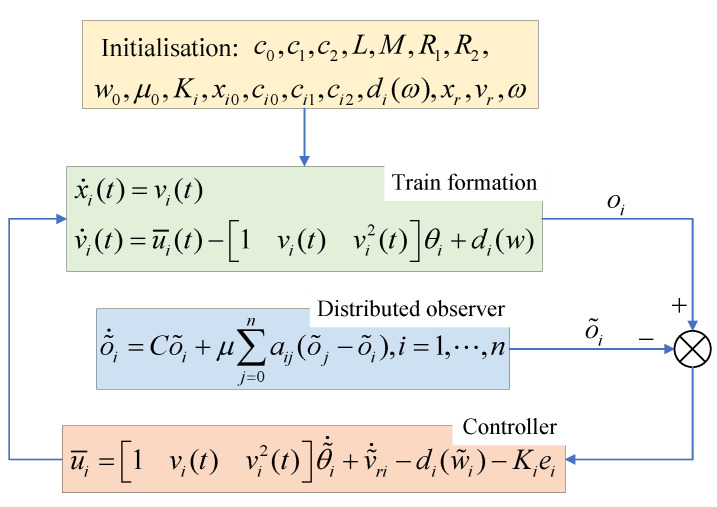
Block diagram of the cooperative-control scheme.

Based on the analysis above, the distributed cooperative controller designed in this paper is proposed as follows:

(11) $$\begin{array}{c} {\bar{u}}_{i}=\left[\begin{array}{ccc} 1 & v_{i}\left(t\right) & v_{i}^{2}\left(t\right) \end{array}\right]{\dot{\hat{\theta }}}_{i}+{\dot{\hat{v}}}_{ri}-d_{i}\left({\hat{w}}_{i}\right)-K_{i}e_{i} \end{array}$$

where ${\dot{\hat{\theta }}}_{i}$ is the derivative of the estimated value of the uncertain parameter vector $\theta_{i}$, ${\dot{\hat{\theta }}}_{i}={\left[\begin{array}{ccc} 1 & v_{i}\left(t\right) & v_{i}^{2}\left(t\right) \end{array}\right]}^{T}e_{i}$; $e_{i}$ represents the velocity error between the current train and the observed reference velocity, $e_{i}=v_{i}-{\hat{v}}_{ri}$; $K_{i}$ is a yet-to-be-designed positive constant, $K_{i}>0$; ${\hat{w}}_{i}$ represents the estimated value of the disturbance factor for train *i*, with ${\hat{w}}_{i}=S_{1}{\hat{o}}_{i}$ and $S_{1}=\left[\begin{array}{ccc} 0 & 0 & 1 \end{array}\right]$; ${\hat{v}}_{ri}$ represents the estimated value of the reference curve for train *i*, where ${\hat{v}}_{ri}=S_{2}{\hat{o}}_{i}-\sum_{j=0}^{n}a_{ij}{▽}_{xi}V\left(d_{ij}\right)$ and $S_{2}=\left[\begin{array}{ccc} 0 & 1 & 0 \end{array}\right]$; ${▽}_{xi}V\left(d_{ij}\right)$ corresponds to the vector in the negative gradient direction of the barrier Lyapunov function $V(d_{ij})$; $a_{ij}$ represents an element in the adjacency matrix of the train-formation communication topology, indicating whether there is a communication link between nodes *i* and *j*. The overall control block diagram can refer to Figure 6.

**Lemma** **2** ([47]). *For any differentiable function $f\left(x,d\right):R^{n}\times R^{l}\to R$, if the function satisfies f(0,d)=0, then there exists a smooth function m(x,d)≥0 such that*

(12) $$\begin{array}{c} \left|f(x,d)|\leq ∥x∥m(x,d\right) \end{array}$$

**Lemma** **3** ([48]). *For any positive constants $k_{a1}$ and $k_{b1}$, we define the open set $Z_{1}:=\{z_{1}\in R|$$-k_{a1}<z_{1}<k_{b1}\}\subset R$ and $N:=R^{l}\times Z_{1}\subset R^{l+1}$. Consider a system $\dot{\eta }=h\left(t,\eta \right)$, where $\eta :={\left[\omega ,z_{1}\right]}^{T}\in N$, and $h:=R_{+}\times Z_{1}\subset R^{l+1}$ is piecewise continuous and uniformly continuous on $R_{+}\times N$, satisfying the local Lipschitz condition. Assume there exist continuous differentiable functions $U:R^{l}\to R_{+}$ and $V_{1}:Z_{1}\to R_{+}$ positive definite on their domains, then we have:*

(13) $$\begin{array}{c} Whenz_{1}\to -k_{a1}orz_{1}\to k_{b1}thenV_{1}\left(z_{1}\right)\to \infty ,\\ \gamma_{1}\left(∥\omega ∥\right)\leq U\left(\omega \right)\leq \gamma_{2}\left(∥\omega ∥\right) \end{array}$$

*where $\gamma_{1}$ and $\gamma_{2}$ are $K_{\infty }$ class functions. Let $V\left(\eta \right):=V_{1}\left(z_{1}\right)+U\left(\omega \right)$ and $z_{1}\left(0\right)\in \left(-k_{a1},k_{b1}\right)$. If the following inequality holds:*

(14) $$\begin{array}{c} \dot{V}=\frac{\partial V}{\partial \eta }h\leq 0 \end{array}$$

*then for all ∀t∈[0,∞), $z_{1}\left(t\right)$ will remain within the open set $\left(-k_{a1},k_{b1}\right)$.*

## 3. Main Results

**Theorem** **1.** *Consider a train formation consisting of n trains as described in (4), with its reference trajectory and disturbances generated by (7), satisfying Assumptions 2–4. Given any maximum communication range $R_{1}>0$ and minimum safe spacing $R_{2}\in \left[0,R_{1}\right)$ between adjacent trains, the designed distributed controller (11) can ensure that the closed-loop system, for given initial states and parameters, maintains the spacing between any adjacent trains within the range $\left(R_{2},R_{1}\right)$ throughout the entire operation and simultaneously satisfies the consistency protocol (8).*

**Proof.**  The global Lyapunov function is chosen as follows:

(15) $$\begin{array}{c} V=\sum_{i=1}^{n}\left(\frac{1}{2}\sum_{j=0}^{n}a_{ij}V\left({\hat{d}}_{ij}\right)\right)+\frac{1}{2}\sum_{i=1}^{n}\left(e_{i}^{2}+{\tilde{\theta }}_{i}^{2}\right)+\frac{1}{2}\int_{0}^{{\tilde{o}}^{2}}\rho \left(r\right)dr \end{array}$$

where $a_{ij}$ represents the elements of the adjacency matrix *A*, which describes the communication relationships among multi-agent trains; ${\hat{d}}_{ij}$ denotes the estimated value of the spacing; $\theta_{i}$ stands for the estimation error of parameters; $\tilde{o}$ signifies the discrepancy between the observed value and the actual value of the external system, where $\tilde{o}={\left[\begin{array}{c} {\tilde{o}}_{1},{\tilde{o}}_{2},\cdots ,{\tilde{o}}_{n}, \end{array}\right]}^{T}$ and ${\tilde{o}}_{i}={\hat{o}}_{1}-o_{i}$; ρ(r) denotes the smooth function to be designed.

From (10), it can be inferred that the barrier Lyapunov function $V(x_{ij})$ is positive definite, hence *V* is positive definite. Then, calculate the derivative of the global Lyapunov function *V* with respect to time *t* as below:

(16) $$\begin{array}{cc} \dot{V}= & \sum_{i=1}^{n}\left(\frac{1}{2}\sum_{j=0}^{n}a_{ij}\left({\dot{\hat{x}}}_{i}\nabla_{x_{i}}V\left({\hat{d}}_{ij}\right)+{\dot{\hat{x}}}_{j}\nabla_{x_{j}}V\left({\hat{d}}_{ij}\right)\right)\right)+\sum_{i=1}^{n}e_{i}{\dot{e}}_{i}+\sum_{i=1}^{n}{\tilde{\theta }}_{i}{\dot{\tilde{\theta }}}_{i}+\rho \left({\tilde{o}}^{2}\right)\tilde{o}\dot{\tilde{o}} \end{array}$$

Due to the estimated spacing ${\hat{d}}_{ij}={\hat{x}}_{i}-{\hat{x}}_{j}-L=\left({\hat{x}}_{i}-{\hat{x}}_{r}\right)-\left({\hat{x}}_{j}-{\hat{x}}_{r}\right)-L=x_{i}-x_{j}-L=d_{ij}$, then for all train i=1,⋯,n within the formation, it can be derive that

(17) $$\begin{array}{c} {\hat{v}}_{ri}=S_{2}{\hat{o}}_{i}-\sum_{j=0}^{n}a_{ij}\nabla_{x_{i}}V\left(d_{ij}\right)=S_{2}{\hat{o}}_{i}-\sum_{j=0}^{n}a_{ij}\nabla_{{\hat{x}}_{i}}V\left({\hat{d}}_{ij}\right) \end{array}$$

By rearranging terms, we have:

(18) $$\begin{array}{cc} \sum_{j=0}^{n}a_{ij}\nabla_{{\hat{\ell }}_{i}}V\left({\hat{d}}_{ij}\right)= & S_{2}{\hat{o}}_{i}-{\hat{v}}_{ri}=S_{2}{\tilde{o}}_{i}+S_{2}o-v_{i}+e_{i}\\ = & S_{2}{\tilde{o}}_{i}+v_{r}-v_{i}+e_{i}=S_{2}{\tilde{o}}_{i}-{\hat{v}}_{i}+e_{i} \end{array}$$

According to symmetry, from (18), we have:

(19) $$\begin{array}{cc} \sum_{i=1}^{n} & \left(\frac{1}{2}\sum_{j=0}^{n}a_{ij}\left({\dot{\hat{x}}}_{i}\nabla_{x_{i}}V\left({\hat{d}}_{ij}\right)+{\dot{\hat{x}}}_{j}\nabla_{x_{j}}V\left({\hat{d}}_{ij}\right)\right)\right)\\ \; & =\sum_{i=1}^{n}\left(\sum_{j=0}^{n}a_{ij}{\hat{v}}_{i}\nabla_{x_{i}}V\left({\hat{d}}_{ij}\right)\right)\\ \; & =\sum_{i=1}^{n}{\hat{v}}_{i}\sum_{j=0}^{n}a_{ij}\nabla_{x_{i}}V\left({\hat{d}}_{ij}\right)\\ \; & =\sum_{i=1}^{n}{\hat{v}}_{i}\left(S_{2}{\tilde{c}}_{i}-{\hat{v}}_{i}+e_{i}\right) \end{array}$$

Continuing with the application of Young’s inequality, we have

(20) $$\begin{array}{cc} \; & \sum_{i=1}^{n}{\hat{v}}_{i}\left(S_{2}{\tilde{o}}_{i}-{\hat{v}}_{i}+e_{i}\right)\\ \; & \leq \sum_{i=1}^{n}\left(\frac{1}{4}{\hat{v}}_{i}^{2}+{∥S_{2}{\tilde{o}}_{i}∥}^{2}+\frac{1}{4}{\hat{v}}_{i}^{2}+e_{i}^{2}-{\hat{v}}_{i}^{2}\right)\\ \; & \leq \sum_{i=1}^{n}\left(-\frac{1}{2}v_{i}^{2}+e_{i}^{2}\right)+{∥S_{2}∥}^{2}{∥\tilde{o}∥}^{2} \end{array}$$

It is evident that ${\dot{e}}_{i}={\dot{v}}_{i}-{\dot{\hat{v}}}_{ri}$ and ${\dot{\tilde{\theta }}}_{i}={\dot{\hat{\theta }}}_{i}-{\dot{\theta }}_{i}={\dot{\hat{\theta }}}_{i}$, then we have

(21) $$\begin{array}{c} \sum_{i=1}^{n}e_{i}{\dot{e}}_{i}+\sum_{i=1}^{n}{\tilde{\theta }}_{i}{\dot{\tilde{\theta }}}_{i}=\sum_{i=1}^{n}e_{i}\left({\dot{v}}_{i}-{\dot{\hat{v}}}_{ri}\right)+\sum_{i=1}^{n}{\tilde{\hat{\theta }}}_{i}{\dot{\hat{\theta }}}_{i} \end{array}$$

Define ${\tilde{\omega }}_{i}={\hat{\omega }}_{i}-\omega$, substituting it into the distributed control law (11), system (5) can be rewritten as

(22) $$\left\{\begin{array}{cc} {\dot{x}}_{i}= & v_{i}\\ {\dot{v}}_{i}= & \left[\begin{array}{ccc} 1 & v_{i} & v_{i}^{2} \end{array}\right]{\tilde{\theta }}_{i}+{\tilde{d}}_{i}\left({\tilde{\omega }}_{i},\omega \right)-K_{i}e_{i}+{\dot{\hat{v}}}_{ri} \end{array}\right.$$

where ${\tilde{d}}_{i}\left({\tilde{\omega }}_{i},\omega \right)=d_{i}\left(\omega \right)-d_{i}\left({\tilde{\omega }}_{i}+\omega \right)$. According to Lemma 2, there exists a smooth function ${\tilde{d}}_{i}\left({\tilde{\omega }}_{i},\omega \right)\geq 0$. Furthermore, there exists a smooth function ${\hat{d}}_{i}\left(\tilde{o}\right)\geq 0$. Thus, for all ω∈R, we have:

(23) $$\begin{array}{c} {\tilde{d}}_{i}^{2}\left({\tilde{\omega }}_{i},\omega \right)\leq {\tilde{\omega }}_{i}^{2}{\bar{d}}_{i}\left({\tilde{\omega }}_{i},\omega \right)\leq {∥\tilde{o}∥}^{2}{\hat{d}}_{i}\left(\tilde{o}\right) \end{array}$$

Continuing with the substitution of (22) into (21), and applying Young’s inequality based on the conclusion derived from (23), we have

(24) $$\begin{array}{cc} \; & \sum_{i=1}^{n}e_{i}{\dot{e}}_{i}+\sum_{i=1}^{n}{\tilde{\theta }}_{i}{\dot{\tilde{\theta }}}_{i}=\sum_{i=1}^{n}e_{i}\left({\dot{v}}_{i}-{\dot{\hat{v}}}_{ri}\right)+\sum_{i=1}^{n}{\tilde{\theta }}_{i}{\dot{\hat{\theta }}}_{i}\\ \; & =\sum_{i=1}^{n}e_{i}\left(\left[\begin{array}{ccc} 1 & v_{i} & v_{i}^{2} \end{array}\right]{\tilde{\theta }}_{i}+{\tilde{d}}_{i}\left({\tilde{\omega }}_{i},\omega \right)-K_{i}e_{i}\right)+\sum_{i=1}^{n}{\tilde{\theta }}_{i}{\left[\begin{array}{ccc} 1 & v_{i} & v_{i}^{2} \end{array}\right]}^{T}e_{i}\\ \; & =\sum_{i=1}^{n}e_{i}\left({\tilde{d}}_{i}\left({\tilde{\omega }}_{i},\omega \right)-K_{i}e_{i}\right)\\ \; & \leq \sum_{i=1}^{n}\left(\frac{1}{4}e_{i}^{2}+{\tilde{d}}_{i}^{2}\left({\tilde{\omega }}_{i},\omega \right)-K_{i}e_{i}^{2}\right)\\ \; & \leq \sum_{i=1}^{n}\left(-\left(K_{i}-\frac{1}{4}\right)e_{i}^{2}\right)+{∥\tilde{\delta }∥}^{2}{\hat{d}}_{i}\left(\tilde{o}\right) \end{array}$$

Based on the definition of $\tilde{o}$, (9) can be rewritten in the following compact form

(25) $$\begin{array}{c} \dot{\tilde{o}}=\left(I_{n}⊗C-\mu L\right)\tilde{o} \end{array}$$

Here, *L* represents the Laplacian matrix of graph *G*. According to Lemma 1, *L* is positive definite. The number of connected subgraphs of the communication topology graph *G* of the train formation is finite. Denoting the set of all connected subgraphs of graph *G* as $\left\{G_{1},G_{2},\cdots ,G_{nG},\right\}$, the corresponding set of Laplacian matrices is given by $\left\{L_{1},L_{2},\cdots ,L_{nG}\right\}$.

Then, it can be derived that

(26) $$\begin{array}{cc} \tilde{o}\tilde{\tilde{o}} & ={\tilde{o}}^{T}\left(I_{n}⊗\left(C+C^{T}\right)-2\mu L\right)\tilde{o}\\ \; & \leq \bar{\lambda }∥\tilde{o}{∥}^{2}-2\mu \underline{\lambda }{∥\tilde{o}∥}^{2} \end{array}$$

where $\bar{\lambda }=\lambda_{\max}\left(C+C^{T}\right)$, $\underline{\lambda }=\min\left\{\lambda_{2}\left(L_{1}\right),\ldots ,\lambda_{2}\left(L_{n_{G}}\right)\right\}$. Choosing $\mu \geq \bar{\lambda }+1/2\underline{\lambda }$, we have

(27) $$\begin{array}{c} \tilde{o}\dot{\tilde{o}}\leq -{∥\tilde{o}∥}^{2} \end{array}$$

Furthermore, it is necessary to choose a suitable function $\rho \left({\tilde{o}}^{2}\right)$ such that it satisfies the following condition:

(28) $$\begin{array}{c} \rho \left(∥\tilde{o}{∥}^{2}\right)\geq \hat{d}\left(\tilde{o}\right)+{∥S_{2}∥}^{2}+1 \end{array}$$

According to (27) and (28), it can be obtained that

(29) $$\begin{array}{c} \rho \left(∥\tilde{o}{∥}^{2}\right)\tilde{o}\tilde{\tilde{o}}\leq -\left(\hat{d}\left(\tilde{o}\right)+{∥S_{2}∥}^{2}+1\right){∥\tilde{\delta }∥}^{2} \end{array}$$

Then, combined with (20), (24) and (29), it can be derived that

(30) $$\begin{array}{cc} \dot{V}\leq & \sum_{i=1}^{n}\left(-\frac{1}{2}v_{i}^{2}+e_{i}^{2}\right)+{∥S_{2}∥}^{2}{∥\tilde{o}∥}^{2}+\sum_{i=1}^{n}\left(-\left(K_{i}-\frac{1}{4}\right)e_{i}^{2}\right)\\ \; & +∥\tilde{o}{∥}^{2}{\hat{d}}_{i}\left(\tilde{o}\right)-\left(\hat{d}\left(\tilde{o}\right)+{∥S_{2}∥}^{2}+1\right){∥\tilde{o}∥}^{2}\\ = & \sum_{i=1}^{n}\left(-\frac{1}{2}v_{i}^{2}-\left(K_{i}-\frac{5}{4}\right)e_{i}^{2}\right)-{∥\tilde{o}∥}^{2} \end{array}$$

Choosing a positive constant $K_{i}\geq 7/4$, we have

(31) $$\begin{array}{c} \dot{V}\leq -\frac{1}{2}\sum_{i=1}^{n}\left(v_{i}^{2}+e_{i}^{2}\right)-{∥\tilde{o}∥}^{2} \end{array}$$

Therefore, it follows that dV(t)/dt≤0. Integrating both sides of this inequality from 0 to *t*, we obtain V(t)−V(0)≤0,∀t≥0. Consequently, we conclude that V(t),∀t≥0 has an upper bound, and its upper bound is V(0). Assuming there exists (i,j)∈ε such that V(t) tends to infinity, it is evident that this contradicts the boundedness of V(t). According to Lemma 3, it can be inferred that $R_{2}<x_{ij}<R_{1}$. In other words, the spacing between adjacent trains within the formation remains within the range of $\left(R_{2},R_{1}\right)$ throughout the entire operation, ensuring that collisions or communication loss do not occur within the formation.

Next, it can be deduced that ${\hat{v}}_{i}=0,dV\left(t\right)/dt=0$ if and only if dV(t)/dt=0. According to the LaSalle Invariance Principle, for the autonomous system (5), every closed-loop trajectory under any initial condition asymptotically converges to the largest invariant set $\left\{\left({\hat{x}}_{i},{\hat{v}}_{i}\right)|dV\left(t\right)/dt=0\right\}$, where

(32) $$\begin{array}{c} \lim_{t\to \infty }{\hat{v}}_{i}=0 \end{array}$$

It can be deduced that any train within the formation can asymptotically converge to the target velocity. Thus, the proof is complete. □

## 4. Simulation and Verification

This simulation case study considers a train formation comprising four trains. The data are sourced from the route data and reference curves of the Guangzhou Metro Line 22, specifically the three stations and two sections from Shiguang Road Station to Chentougang Station. The speed-convergence phases of the train formation in the segments from Shiguang Road Station to Guangzhou South Station (referred to as Section One) and from Guangzhou South Station to Chentougang Station (referred to as Section Two) are separately examined. The relevant parameter settings for the simulation case are as follows: all initial speeds of trains within the formation are set to zero; the coefficients of the Davis equations in the dynamic models of the trains within the formation are $c_{0}=9.888$, $c_{1}=0.05$, and $c_{2}=0.00195$; the length of each train is L=180 m; the mass of each train when empty is M=378.081t; the minimum safe distance between adjacent trains within the formation is $R_{2}=60$ m, and the maximum communication distance is $R_{1}=80$ m; the initial value of the disturbance factor is $w_{0}=0$; the parameter in the distributed observer (9) is $\mu_{0}=10$; the control gain parameters for the proposed distributed controller (10) are $K_{i}=15$. Additional initial parameter settings for each train are provided in Table 1. The distribution disturbance $d_{i}\left(\omega \right)$ experienced by each train and the initial parameter settings for the distributed observer are detailed in Table 2.

**Figure 7:**
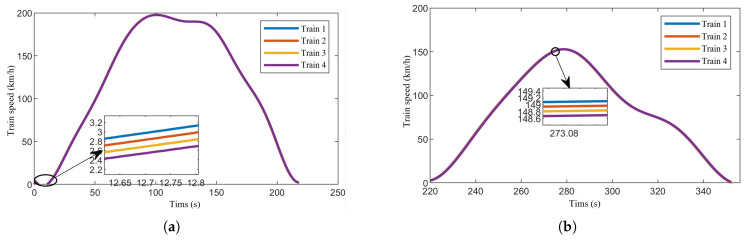
Speed-time curves of the train formation. (**a**) Speed of train formation in Section One. (**b**) Speed of train formation in Section Two.

**Figure 8:**
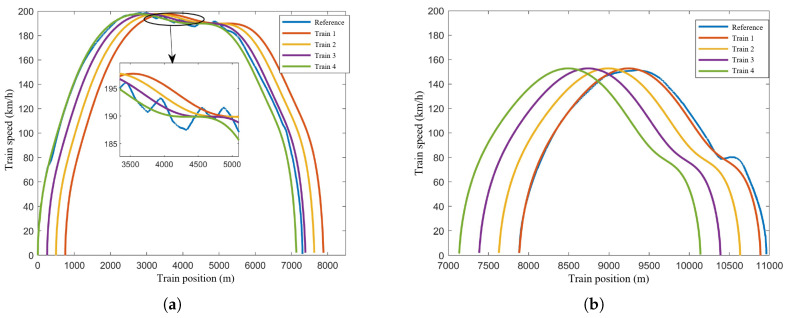
Speed-position curves of the train formation. (**a**) Speed-position curve of the train formation in Section One. (**b**) Speed-position curve of the train formation in Section Two.

**Figure 9:**
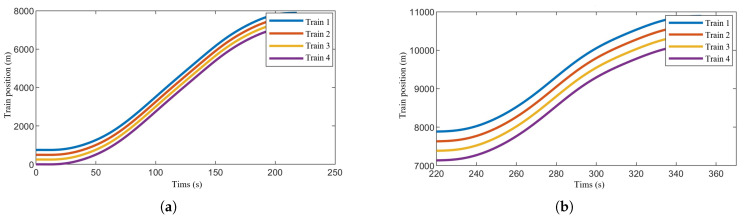
Positionof the train formation. (**a**) Position of the train formation in Section One. (**b**) Position of the train formation in Section Two.

Figure 7, Figure 8 and Figure 9 show the speed-time, speed-position, and trajectory curves, respectively, for the train formation during operation. It is evident that the trains quickly converge to a uniform speed and maintain stable operation. Both speed tracking and position tracking exhibit excellent performance. Additionally, no collisions occurred between adjacent trains within the sections, ensuring safety throughout the operation.

**Figure 10:**
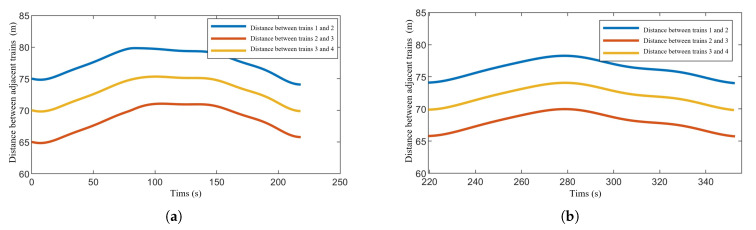
Distances between neighboring trains in train formation during operation. (**a**) Distances between neighboring trains in train formation during operation in Section One. (**b**) Distances between neighboring trains in train formation during operation in Section Two.

Figure 10 illustrates the distances between adjacent trains during operation. It can be observed that under the proposed distributed controller, the inter-train distances are maintained within the preset range of 60 m to 80 m.

**Figure 11:**
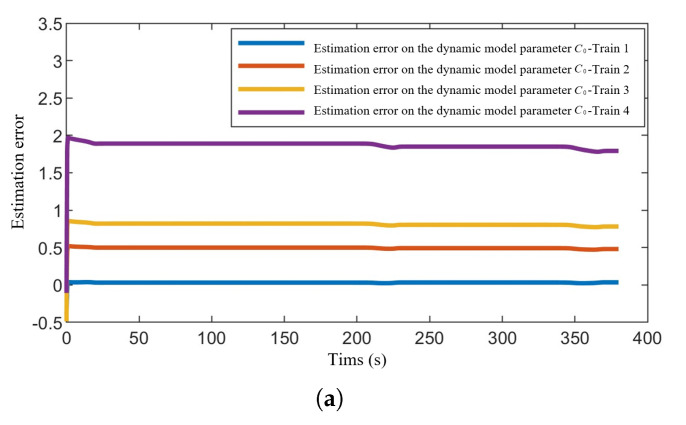

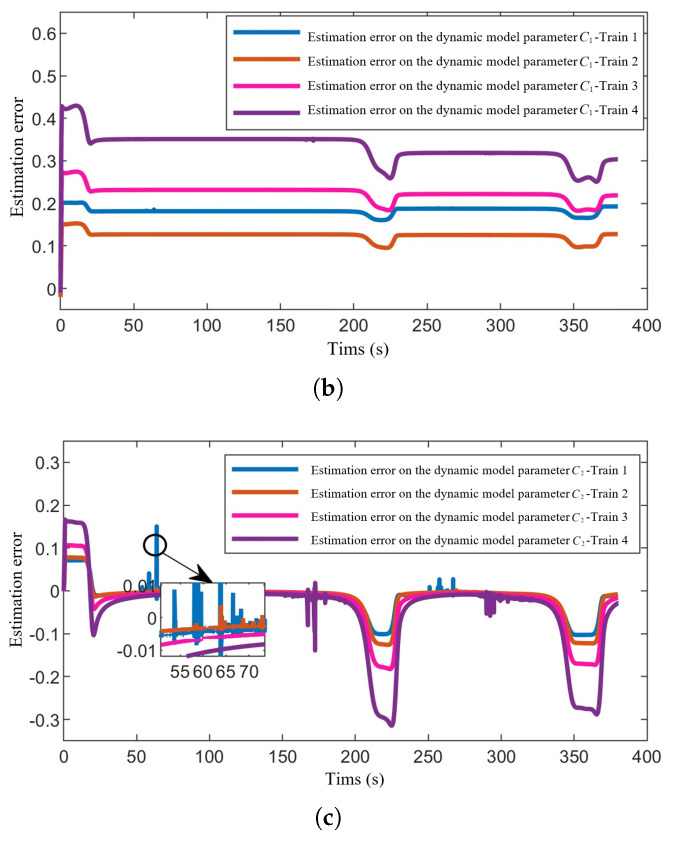
Estimation errors of train dynamics model parameters for each train. (**a**) Estimation errors of parameter $c_{0}$ in train dynamics model. (**b**) Estimation errors of parameter $c_{1}$ in train dynamics model. (**c**) Estimation errors of parameter $c_{2}$ in train dynamics model.

Figure 11 presents the estimation errors of the dynamic model parameters for each train within the formation. It can be observed that only the estimation error of parameter $C_{3}$ has essentially converged to 0, while the estimation errors for parameters $C_{1}$ and $C_{2}$ have not fully converged. Additionally, by comparing Figure 11a–c, it is evident that the estimation accuracy for parameters $C_{3}$, $C_{2}$, and $C_{1}$ degrades in that order. Similarly, for the same figure, the estimation performance for Train 1, Train 2, Train 3, and Train 4 also deteriorates sequentially. For the former observation, this is primarily because the amount of information that can be obtained from system dynamics for estimating parameters $C_{3}$, $C_{2}$, and $C_{1}$ decreases progressively (see ${\hat{\theta }}_{i}$). The impact of system state changes on parameter estimation weakens sequentially (as judged by the magnitude of curve changes in Figure 11a–c). Especially for parameter $C_{1}$, which can only be estimated through *e*, the information required for estimating $C_{1}$ is severely insufficient, resulting in the poorest estimation accuracy for $C_{1}$. Regarding the latter observation, since the train behind two adjacent trains can only obtain state information from the front train, the signal-to-noise ratio of the information transmission decreases due to the influence of system parameters and external disturbances. Consequently, Train 1 receives more useful state information compared to Train 4, leading to a sequential degradation in the estimation performance from Train 1 to Train 4. Overall, the proposed distributed adaptive control algorithm effectively estimates the unknown parameters for each train, ensuring system stability through real-time compensation despite model parameter uncertainties.

**Figure 12:**
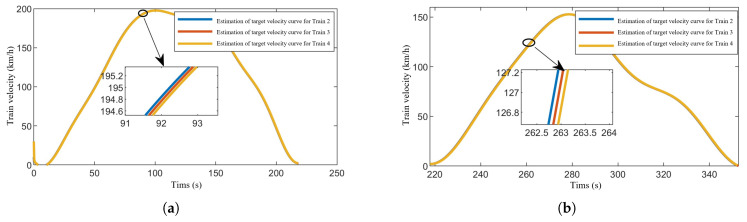

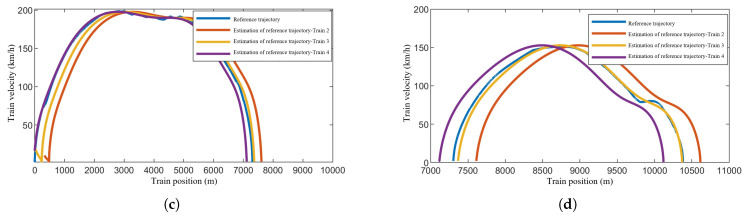
The simulation results of distributed observer. (**a**) Estimation of target speed curve of Section One. (**b**) Estimation of target speed curve of Section One. (**c**) Estimation of reference speed-position curve of Section One. (**d**) Estimation of reference speed-position curve of Section Two.

Figure 12 presents the simulation results of the proposed distributed observer in this section. As the lead train directly receives the reference trajectory, distributed observers are utilized for trajectory estimation among all following trains within the formation, excluding the lead train. Figure 12a–d, respectively, demonstrate the estimation effects of the target speed curve and the reference trajectory curve for each following train. The distributed observer enables accurate estimation of the reference trajectory for each following train within the train formation, ensuring effective control.

**Figure 13:**
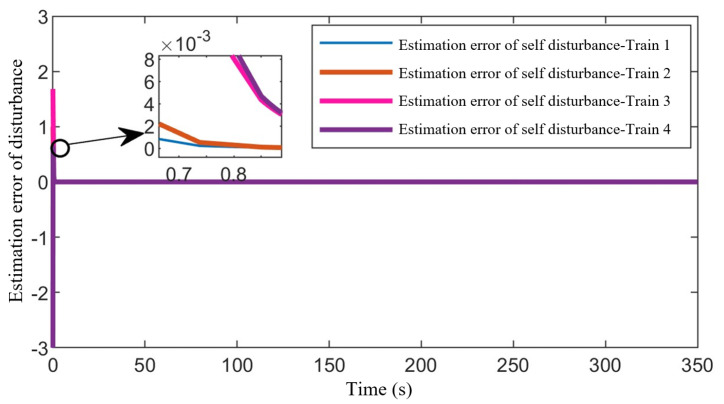
Estimation error of the disturbance.

Figure 13 illustrates the estimation effects of disturbances for each train within the formation using the distributed observer. It can be observed from the graph that the estimation errors of disturbances for each train converge rapidly to zero within a short period. This simulation result validates the effectiveness of the proposed distributed observer, which ensures the stability of the train-formation control by compensating for external system disturbances.

## 5. Conclusions

In response to the high safety requirements for train-formation operation, this paper addresses the maintenance of adjacent train spacing during the speed-convergence phase of train formation as the objective. The coordinated control problem of train formation is modeled as a leader-following aggregation problem with parameter and disturbance uncertainties. Corresponding controller design methods are proposed. Considering that not all trains in the train formation can receive reference trajectory information during operation and various noise disturbances exist, a distributed observer is designed in this paper, which only requires information from neighboring trains. Furthermore, to ensure the safe maintenance of train-formation spacing, a cooperative-control algorithm based on barrier Lyapunov function is designed to enforce constraint preservation. This algorithm integrates nonlinear adaptive control to handle parameter uncertainties in the train-formation model, and the stability of the algorithm is proven through Lyapunov stability criteria. Finally, a practical example is provided to further demonstrate the effectiveness and robustness of the cooperative-control method.

## Figures and Tables

**Table 1:** Initial parameter setting of each train in formation.

Train Number *i*	Initial Position $x_{i0}$ (m)	Initial Estimation Parameters of Train Dynamics Model		
		${\hat{c}}_{i0}$	${\hat{c}}_{i1}$	${\hat{c}}_{i2}$
1	750	9.5	0.1	0.002
2	495	9.8	0.03	0.005
3	250	9.4	0.056	0.003
4	0	9.78	0.04	0.0015

**Table 2:** Initial parameter setting of each train in formation.

Train Number *i*	Disturbance Distribution $d_{i}\left(\omega \right)$	Initial Estimation Parameters of Distributed Observers ${\hat{o}}_{i0}$		
		${\hat{x}}_{r}$ **(m)**	${\hat{v}}_{r}$ **(km/h)**	$\hat{\omega }$
1	$\omega^{2}$	-	-	−2
2	$\omega^{3}$	325	10	0
3	sin(ω)	100	30	−1
4	$\omega^{2}$	−50	30	2

## Data Availability

The data presented in this study are available on request from the corresponding author.
